# Investigating Differences across Host Species and Scales to Explain the Distribution of the Amphibian Pathogen *Batrachochytrium dendrobatidis*

**DOI:** 10.1371/journal.pone.0107441

**Published:** 2014-09-15

**Authors:** Anna C. Peterson, Valerie J. McKenzie

**Affiliations:** 1 Ecology and Evolutionary Biology, University of Colorado, Boulder, Colorado, United States of America; 2 Ecology and Evolutionary Biology, Tulane University, New Orleans, Louisiana, United States of America; Imperial College Faculty of Medicine, United Kingdom

## Abstract

Many pathogens infect more than one host species, and clarifying how these different hosts contribute to pathogen dynamics can facilitate the management of pathogens and can lend insight into the functioning of pathogens in ecosystems. In this study, we investigated a suite of native and non-native amphibian hosts of the pathogen *Batrachochytrium dendrobatidis* (Bd) across multiple scales to identify potential mechanisms that may drive infection patterns in the Colorado study system. Specifically, we aimed to determine if: 1) amphibian populations vary in Bd infection across the landscape, 2) amphibian community composition predicts infection (e.g., does the presence or abundance of any particular species influence infection in others?), 3) amphibian species vary in their ability to produce infectious zoospores in a laboratory infection, 4) heterogeneity in host ability observed in the laboratory scales to predict patterns of Bd prevalence in the landscape. We found that non-native North American bullfrogs (*Lithobates catesbeianus*) are widespread and have the highest prevalence of Bd infection relative to the other native species in the landscape. Additionally, infection in some native species appears to be related to the density of sympatric *L. catesbeianus* populations. At the smaller host scale, we found that *L. catesbeianus* produces more of the infective zoospore stage relative to some native species, but that this zoospore output does not scale to predict infection in sympatric wild populations of native species. Rather, landscape level infection relates most strongly to density of hosts at a wetland as well as abiotic factors. While non-native *L. catesbeianus* have high levels of Bd infection in the Colorado Front Range system, we also identified Bd infection in a number of native amphibian populations allopatric with *L. catesbeianus*, suggesting that multiple host species are important contributors to the dynamics of the Bd pathogen in this landscape.

## Introduction

Emerging infectious diseases are an increasing threat to the health of both human and wildlife populations, with many pathogens of concern infecting more than one host species (e.g., [1], [2]). Hosts may vary in their responses to infection, and heterogeneity of hosts in their mortality rates, ability to obtain, maintain and transmit pathogens has important consequences for pathogen dynamics and disease outcomes in ecological communities (e.g., [3], [4], [5], [6]). It is important to investigate multiple traits across species and scales; as, for example, rare species have been found to be the most important hosts contributing to pathogen transmission in some systems (e.g., [5], [6]), while abundant species are important in others (e.g., [4]). Intrinsic characteristics of host species, in addition to population and landscape level characteristics of host populations, relate to the ability of some host species to contribute more to pathogen persistence and transmission in the landscape relative to other hosts [7]. At the local scale, intraspecific variability within hosts can strongly influence disease dynamics at a larger landscape scale (e.g., [8]), further highlighting the importance of understanding individual and species-level variation to aid in interpreting landscape-level patterns of pathogen presence or prevalence.

The fungal pathogen *Batrachochytrium dendrobatidis* (hereafter referred to as Bd) is an emerging infectious disease linked to amphibian declines globally. The pathogen Bd is transmitted directly between individuals through a flagellated free-swimming zoospore stage and causes the disease chytridiomycosis, which results from the disruption of cutaneous osmoregulation and, in some cases, leads to cardiac failure and ultimately death of hosts [9], [10]. However, there appears to be a gradient of susceptibility to the disease, with some species showing clear mortality in the lab and massive declines in the wild as a result of infection with Bd (e.g., [11], [12], [13], [14], [15], [16]. Some other species show little evidence of disease driven morbidity or mortality in the lab or in the field, even in regions where other species are undergoing declines [11], [12], [13], [14], [15], [16]. One such species that has shown little mortality when infected with Bd is the North American bullfrog (*Lithobates catesbeianus*) [14]. This species has been introduced nearly globally [17], including in Colorado (and other regions of the western US), where the first records of this species date back to the 1940’s [18]. The global trade in North American bullfrogs has played an important role in facilitating the long-distance transport of Bd [19], [20], [21] potentially facilitating the hybridization of once disparate Bd lineages [22], [23]. This hybridization is hypothesized to be one potential mechanism responsible for the emergence of the virulent and widespread Global Panzootic Lineage (Bd-GPL) Bd strain [23], [24]. Due to the wide distribution of *L. catesbeianus* populations, as well as the lack of pathology associated with Bd infection in this species, bullfrogs have been identified as a potential reservoir for Bd [14], [21], [24]. While the role of *L. catesbeianus* as a global-trade transport host has been well supported [21], [22], [23], there is a lack of information regarding how this species may influence pathogen dynamics in the landscape, particularly in areas where they have been introduced and are well established. As native species in the regions where *L. catesbeianus* populations have been introduced have variable tolerance to chytridiomycosis, *L. catesbeianus* populations have the potential to strongly impact Bd dynamics in susceptible species in these areas where they are introduced.

**Figure 1:**
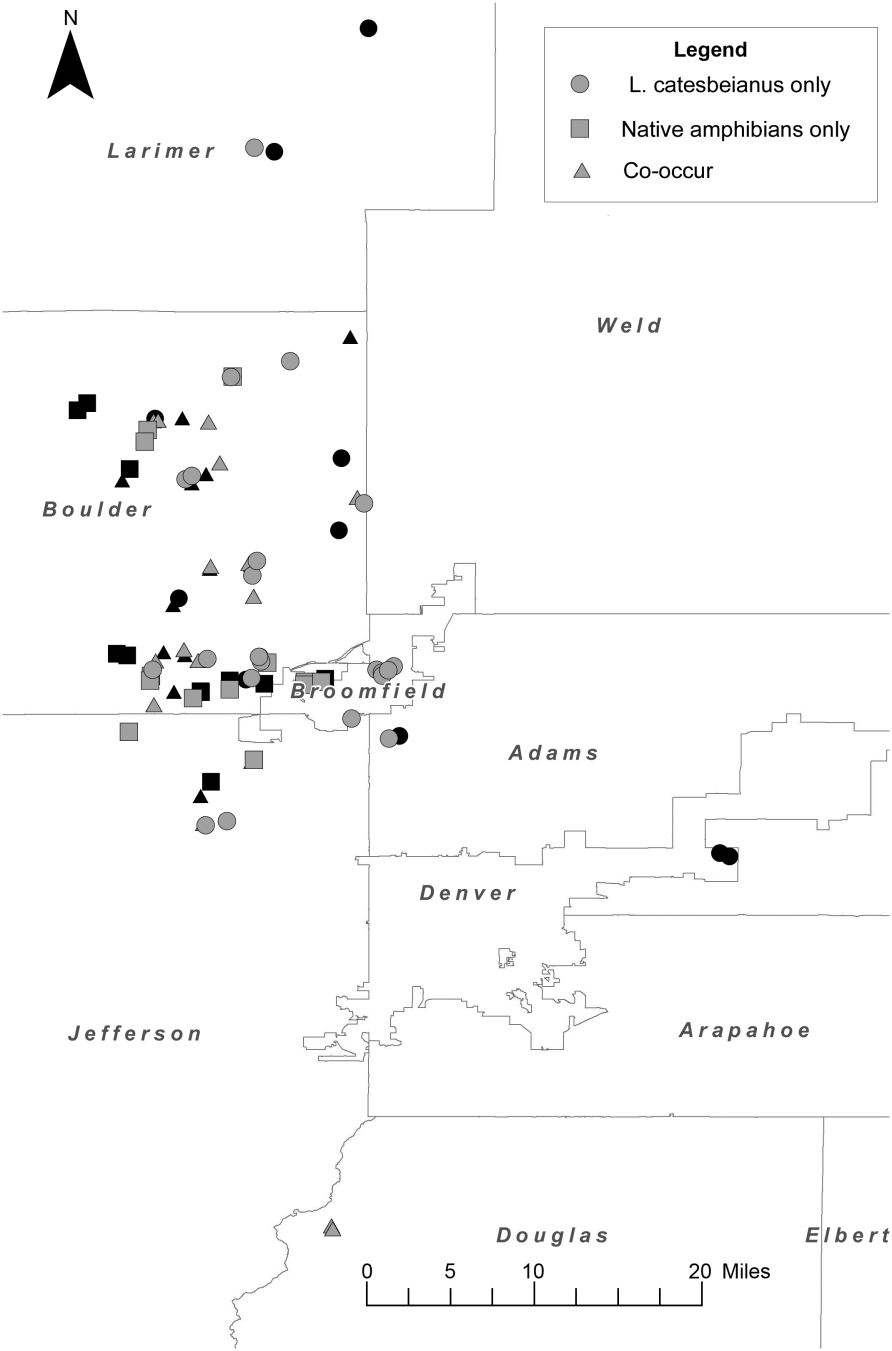
Map of all wetlands included in the Colorado Front Range amphibian survey. All 99 wetlands at which we sampled for amphibians are included above. Wetlands at which we collected population level Bd data (n = 36) are in black, all other wetlands are in grey (n = 63). Circles (*L. catesbeianus* only) represent wetlands where we detected only *L. catesbeianus* individuals, squares represent wetlands where we detected populations of at least one native amphibian species, and triangles represent wetlands where we detected sympatric populations of at least one native amphibian species and *L. catesbeianus*.

The Colorado Front Range, the high plains region immediately east of the Rocky Mountains (Figure 1), is an excellent system to explore the role of invasive *L. catesbeianus* in the distribution of Bd at the landscape scale relative to native species. The Colorado Front Range region of Colorado is east of, and below, the high elevation areas where boreal toad (*Anaxyrus boreas*) declines due to Bd have been ongoing for the last few decades [25], [26]. Historically, the Front Range region was dominated by grasslands and small, ephemeral water bodies that supported large populations of native Northern leopard frogs (*Lithobates pipiens*) as well as other native amphibians including tiger salamanders (*Ambystoma tigrinum*), Western chorus frogs (*Pseudacris triseriata*), and Woodhouse’s toads (*Anaxyrus woodhousii*) [18]. Following land conversion to agriculture and a rapid increase in suburbanization since the 1980s, artificially permanent water bodies in the form of retention ponds, cattle ponds, golf course hazard ponds, and ornamental suburban ponds have replaced natural amphibian habitats [27]. The life history of *L. catesbeianus* in Colorado involves overwintering in the larval stage, and thus, they require permanent water bodies for successful development [18]. The increase in the availability of permanent water bodies and the density of those water bodies in the landscape are linked to the spread of invasive *L. catesbeianus* in the Colorado Front Range [28]. The abundance of invasive *L. catesbeianus* in the Front Range is staggering; a recent study identified populations of *L. catesbeianus* in nearly 50% of 243 wetlands surveyed in the region [28]. Additional work in this system has identified that the degree of urbanization surrounding wetlands is strongly negatively related to vertebrate richness, though invasive *L. catesbeianus* are still highly abundant in these urbanized sites [29]. A re-survey of historical native Northern leopard frog (*L. pipiens*) sites found that the Front Range region had only 2% of sites still supporting leopard frogs, compared with 52% of sites in the Western Slope region (west of the Rockies) [30]. Additionally, the presence of declining *L. pipiens* populations was negatively related to the presence of *L. catesbeianus* populations, further implicating invasive *L. catesbeianus* as a factor related to native amphibian occupancy in the Front Range region [30].

Whether Bd, has played a role in the decline of Northern leopard frogs or other native species in the Front Range remains elusive. Prevalence of Bd is variable but consistently positive across regional populations of all amphibians in Colorado [17], [26]. It appears that several remaining native species are possibly persisting with Bd. This raises the question of how multiple host species contribute to the enzootic disease landscape after the pathogen has been well established. In some systems, native species are capable of tolerating Bd infection and serving as reservoirs relative to more susceptible amphibians, such as Pacific chorus frogs (*Pseudacris regilla*) in the high elevation Sierra Nevada mountains [11]. While North American bullfrogs have also been implicated as potential reservoirs for Bd [14], [21], [24], outside of the global transport systems, this idea has not been well tested in a landscape context. The questions remains, once Bd is well established and has entered the enzootic disease phase (such as in the Colorado Front Range) are different amphibian species relatively equal in their Bd contributions to the environment, or do certain species contribute more and potentially act as a pathogen reservoir to maintain the pathogen in the environment? We hope to address this question in the Colorado Front Range system by determining if: 1) amphibian populations vary in Bd infection across the landscape, 2) amphibian community composition predicts infection (e.g., does the presence or abundance of any particular species influence infection in others?), 3) amphibian species vary in their ability to produce infectious zoospores in a laboratory infection, 4) heterogeneity in host ability observed in the lab scales to predict patterns of Bd prevalence in the landscape.

In our study, we investigated heterogeneity in the intra- and inter-specific responses of multiple amphibian host species to Bd-GPL infection using both field and laboratory approaches. We conducted a field survey in order to determine patterns of amphibian occurrence across the landscape, and also to quantify Bd infection in native amphibian populations found in sympatric and allopatric wetlands with *L. catesbeianus.* By investigating patterns of Bd infection across communities we aimed to clarify if non-native *L. catesbeianus* may be influencing Bd dynamics in native amphibian populations, or vice versa. For the laboratory experiment we isolated a local strain of Bd-GPL from an *L. catesbeianus* individual and used the strain to infect locally collected native amphibian species and non-native *L. catesbeianus* to determine the relative production of the infectious zoospore stage by different hosts over time. We then are able to take information from both the laboratory study to determine if the species level responses to Bd infection observed in the laboratory scale to predict landscape level patterns of Bd infection observed across different populations and communities. By investigating host responses to infection at multiple scales, we are able to identify particular hosts species that may contribute more to Bd dynamics relative to other hosts in the system while also disentangling potential mechanisms that may be driving the differences in infection prevalence across varying amphibian communities. This information is especially important given the devastating nature of Bd (e.g., [31], [32]) and the widespread distribution of invasive *L. catesbeianus* populations [17]. Such approaches may facilitate mitigating the impact of these two factors on declining amphibian communities, and can also be applied to other pathogen/hosts systems.

## Methods

### Ethics statement

All laboratory experiments and field studies were approved by the University of Colorado Institutional Animal Care and Use Committee (protocols 1104.04 and 1108.05) and Bd isolation approved by the University of Colorado Institutional Biosafety Committee (permit application number: BA11-EBIO-McK-01). Care was taken to minimize suffering to experimentally infected animals, and individuals showing symptoms of advanced stages of chytridiomycosis were humanely euthanized. No animals were harmed in the field survey. All access to private property was granted in person to ACP by landowners and/or golf course managers. All access to wetlands located on public open space property was permitted by City of Boulder Open Space and Mountain Parks, Boulder County Parks and Open Space, and Jefferson County Open Space.

### Field survey

During the period of June-August 2011, we surveyed 99 wetlands across 6 counties in the Front Range region of Colorado (Figure 1). To obtain general trends in amphibian species occurrence across this landscape supplemental to previous work in the Colorado Front Range system (e.g. [17], [28], [29]) we surveyed 63 haphazardly selected wetlands to represent common Colorado Front Range wetlands. Using past survey information (e.g. [28], [30], [33]) we preferentially selected 36 wetlands to collect population-level Bd data from wetlands supporting large populations of North American bullfrogs (*L. catesbeianus*), large native amphibian populations, or sympatric populations of native amphibians and *L. catesbeianus* in the same wetland (see Figure 1). We collected Bd samples from amphibians encountered at all 99 wetlands included in the survey, though only Bd data from the 36 wetlands where we collected thorough population-level estimates are presented here. Additionally, we encountered only one breeding population of Northern leopard frogs (*L. pipiens)*, and due to this small sample size Bd data obtained from this population were not included in any further analyses. We obtained population-level Bd infection data from 11 wetlands where we detected only *L. catesbeianus* populations, 14 wetlands where we detected sympatric populations of *L. catesbeianus* and at least one other native amphibian species, and from 11 wetlands where we detected at least one native amphibian species but no *L. catesbeianus* populations.

We collected observations on abiotic as well as biotic characteristics at each wetland included in the survey. To determine the hydro period of a wetland (categorized as either permanent or temporary) we paired on- the- ground field observations, information provided by local management agencies (for wetlands located on Open Space Properties), conversations with private landowners, and Google Earth™ imaging (as in [28], [29]). There are a roughly 8–10 high quality Google Earth™ satellite images available of the Colorado Front Range landscape within the past 2–4 years, and these images were taken across multiple seasons during these years. Using the historical imagery tool we were able to scan all recent historical satellite images of a wetland and view each wetland in summer, fall and winter seasons. We categorized a wetland as temporary if satellite imagery showed that it was dry during any past season, we observed the wetland dry at any point during field sampling, or we were told by management agencies or private landowner that the wetland was a temporary wetland. We categorized all other wetlands as permanent wetlands. To collect information about the biotic factors at each wetland, we used a combination of visual encounter surveys (VES), dip net sweeps and seine net sweeps to detect the presence of amphibian species at each wetland. The VES was conducted immediately upon arrival to a wetland, and was done by walking the perimeter of each wetland and noting the species and number of any amphibians seen or heard within 3 meters of the shoreline, including larval and adult stages. During the VES, we also calculated the coordinates, elevation and area of each wetland using a handheld Garmin GPS 60CSx unit. Following the VES, we completed a total of 10 dip net sweeps at regular intervals around the shoreline by pulling a 1.4 mm mesh size net in a 1.5 meter line perpendicular to shoreline (as per [28], [30]). We placed all contents of the sweep into a plastic tray and recorded the number and species of all amphibians captured, as well as the number of all fish and crayfish captured. Whenever possible, we completed 3–4 seine net hauls by pulling a 0.8×2.0 meter seine net through the water, and recorded the distance of each sweep as well as the number and species of all amphibians captured and the number of all fish and crayfish captured in each seine haul. Due to the difficulty of accurately measuring density of different species of amphibians at different life stages in the field, we combined information from three different estimates of amphibian populations size: VES counts of adult amphibians, counts of larval populations from seine net sweeps, and counts of metamorphosed individuals and larval stages captured in the dip net sweeps. We then added the counts of individuals of each species from these three estimates of population size and divided the size of each population by the area of the wetland in order to obtain an estimate of the density of each species encountered at a wetland.

Adult or recently metamorphosed amphibians captured in the seine net and dip net sweeps, and met our life stage criteria (described below), were swabbed with a sterile cotton tipped swab 25 times on the ventral surface and 5 times on each foot [34]. We swabbed tadpoles 25 times on their mouthparts [34]. Following swabbing, we released all individuals unharmed back into the wetland from which they were captured. At the subset of 36 wetlands where we collected population-level Bd data, after completing the standardized seine net and dip net sweeps, we conducted additional seine net sweeps, dip net sweeps and hand-captures to obtain at least 25 individuals of all species encountered at each wetland (when possible) for swabbing.

To ensure that all individuals included in this study originated from the wetland sampled, we collected Bd samples from only late stage tadpoles or recently metamorphosed individuals. For individuals of the species *L. catesbeianus* and *Ambystoma tigrinum* (tiger salamander), we concentrated sampling on either late stage larvae (Gosner stage 41–46) or recently metamorphosed individuals, as these are the life-stages most feasible to capture in large numbers and Bd detection on these life stages has been shown to be reliable [35], [36], [37]. For individuals of the species *Pseudacris triseriata* (Western chorus frog) and *Anaxyrus woodhousii* (Woodhouse’s toad) we targeted only recently metamorphosed individuals for sampling as larvae of these species have small keratinized mouthparts, limiting areas of potential infection by *Bd* (and thus potentially detectability of the pathogen) [35], [36]. We obtained population-level Bd data from *Lithobates pipiens* (Northern leopard frog) at only one wetland, and we collected swab samples from Gosner Stage 41 tadpoles at this wetland. After collection, we placed all swabs in a cooler and then froze them immediately upon return to the University of Colorado, Boulder. To minimize contamination, all field personnel wore Nitrile gloves when handling amphibians, and changed gloves between the handling and swabbing of each individual. Additionally, we sanitized all seine nets, dip nets, waders and other equipment with a 5–10% bleach solution after completion of sampling at each wetland and let all equipment sun-dry between sampling efforts.

We kept all swabs frozen at −20°C until DNA extraction. We extracted DNA from all swabs using PrepMan Ultra sample preparation reagent, diluted each sample 1/10, and tested all samples for Bd in duplicate using the qPCR protocol outlined in [37]. The average of the two duplicate runs was taken for each individual. If there was greater than one order of magnitude difference in the quantitative readings between duplicate samples, DNA samples were thoroughly re-mixed (30 second vortex and spin x 3) and re-run. In all cases, vortexing and re-running a sample was sufficient to clarify any disagreement between duplicates from the same individual. In all qPCR analyses, we considered any samples with quantitative readings below our lowest standard (1.0 DNA copy) as 0. We used TaqMan Exogenous Internal Positive Control to verify all negative samples represented true zeros, and were not a product of inhibition of the PCR process. About 8% of samples suggested inhibition, and these samples were diluted 1/100 and run a second time with TaqMan Exogenous Internal Positive Control, which showed that our dilution was sufficient to resolve sample inhibition issues.

### Bd isolation

We isolated a local strain of Bd for use in the laboratory portion of this experiment by collecting fifteen (40–44 Gosner stage) *L. catesbeianus* tadpoles from a Boulder county wetland previously identified as having a high prevalence of Bd infection. We focused on collecting individuals that appeared to have mouthpart depigmentation to increase the probability of collecting a Bd infected individual. We returned these tadpoles to the University of Colorado, Boulder and placed them in individual containers and screened each individual for the pathogen Bd by swabbing them on their mouthparts and using the DNA extraction and qPCR methods described above and in [37]. We euthanized infected individuals, removed their mouthparts and placed them onto antibiotic-containing Tryptone plates in accordance with [9]. We placed one large successful colony of Bd sporangia in liquid Tryptone + Gelatin Hydrosylate broth with antibiotics and passaged the culture 2 times, then transferred the culture to Tryptone + Gelatin Hydrosylate broth without antibiotics. The culture was passaged 3 more times before infecting the experimental animals. Four samples of this *Bd* strain were extracted using the Qiagen DNeasy Blood and Tissue kit and sent to Dr. Matthew Fisher’s lab at Imperial College in London. They performed qPCR using primers designed to differentiate Bd-GPL from non-GPL strains (Fisher, *unpublished technique*). Results confirmed that the samples from Colorado bullfrogs are within the Bd-GPL group (Fisher, *personal communication*).

### Laboratory infection study

In order to determine the relative zoospore output of different amphibian species over time, we collected recently metamorphosed and Gosner stage 42–44 *L. catesbeianus*, recently metamorphosed Western chorus frogs (*P. triseriata)*, recently metamorphosed Woodhouse’s toads (*A. woodhousii)* and tiger salamander (*A. tigrinum)* larvae from wetlands located in Boulder county and brought them to the laboratory at the University of Colorado, Boulder. The amphibians were placed in individual containers of a volume relative to their size and kept in a temperature-controlled room at 20°C on a 12-hour light and dark cycle. To control for differences in Bd infection that may occur between amphibian life stages, we concentrated on collecting only recently metamorphosed individuals, though no recently metamorphosed *A. tigrinum* individuals were found, and thus we collected late stage *A. tigrinum* larvae.

Upon return to the lab we allowed individuals 3 days to acclimate, with the exception of *A. tigrinum* individuals, which we maintained in the lab uninfected until they completed metamorphosis. Upon arrival to the lab we screened all individuals for Bd and weighed and measured each individual. Uninfected individuals of each species were split into two groups, one of which was infected with a low dose of our isolated Bd-GPL strain (∼10,000 zoospores), and the other group was infected with a high dose of Bd (∼200,000 zoospores) [16]. We infected all individuals by placing each individual of each species in a container with enough Holtfreter’s solution to cover their bodies, and then added ∼10,000 or ∼200,000 zoospores (as counted with a hemocytometer) from our Boulder County Bd-GPL strain. We left all amphibians in their individual infection containers for 24 hours, and then placed them in individual housing containers with 20–150 ml of Holtfreter’s solution, depending on species. There was some mortality in individuals within the first 0–3 days of the infection, and this was likely due to difficulty of maintaining very small recently metamorphosed individuals in the lab, as all dead individuals were swabbed post-mortem and showed low or no Bd infection. All individuals were swabbed 3-days post infection to determine infection status. Some *L. catesbeianus*, *A. tigrinum* and *P. triseriata* individuals did not test positive for Bd infection after 6-days post infection (see Table S1 in File S1). These individuals were re-infected with the same does of Bd and were screened again 3 and 6 days post infection. A second Bd exposure was sufficient to infect all *L. catesbeianus* individuals, but did not induce infection in all *A. tigrinum* or *P. triseriata* individuals (Table S1 in File S1). Any individuals that remained uninfected after a second infection exposure were removed from the study. In total 8 high treatment *A. tigrinum*, 6 low and 6 high treatment *P. triseriata*, 11 low treatment and 10 high treatment *L. catesbeianus,* 14 low treatment and 11 high treatment *A. woodhousii* were included in this study (Table S1 in File S1).

Beginning 3 days post infection, we removed all species from their individual housing containers and placed each individual into a small plastic container with enough Holtfreter’s solution to cover their bodies (either 100 ml, 50 ml, or 15 ml, depending on species) for 15 minutes. After 15 minutes we removed individuals from their soak container and immediately filtered the Holtfreter’s solution through a Millex-HA 0.45 um filter [11] to capture Bd zoospores released into the solution over the course of the 15-minute soak. This process was repeated once every 3 days for two weeks, after which we reduced the frequency of soaks to once every 4 days for a period of 2 weeks, and then reduced the soak frequency again to once every 5 days for two weeks. Individuals were weighed and measured at least two other times during the course of the experiment, and monitored on a daily basis for symptoms of chytridiomycosis. We terminated the project 62 days post-infection, and weighed and measured each individual at the end of the experiment. In total, all individuals that survived the entire experiment were soaked 15 times over the course of the 62-day experiment. We used PrepMan Ultra sample preparation reagent to extract DNA from all of the filters, diluted each sample 1/10, and ran each sample in duplicate using real time quantitative PCR to determine the number of DNA copies present on each filter [11], [37]. As with the field- collected samples, we considered any filter sample with quantitative readings below our lowest standard (1.0 DNA copy) as 0 and followed the same protocol described previously for any duplicates with quantitative readings that were not within one order of magnitude of each other. We used TaqMan Exogenous Internal Positive Control to verify that all negative samples represented true zeros, and were not a product of inhibition of the PCR process. None of the laboratory collected samples showed inhibition.

### Statistical analyses of field survey data

We used a generalized linear mixed effects model (GLMM) with a binomial error distribution to determine the suite of biotic and abiotic factors that best predict the 1/0 (infected/not infected) status of each individual sampled for Bd in our field survey. We tested all predictor variables for collinearity, and none was found. We created two broad categories of variables (abiotic fixed effects, biotic fixed effects) and created a set of models to predict the infection status of individuals (Table S2 in File S1). Wetland site was included as a random effect in all models. Included in our abiotic category of fixed-effect predictor variables were: wetland area, elevation and wetland hydro period (either permanent or temporary). Included in the biotic category of fixed-effect predictor variables were: the density of *A. tigrinum*, density of *L. catesbeianus*, density of *A. woodhousii*, density of *P. triseriata* and the number of species at a wetland. We used the glmmADMB package in R [38] with a binomial family to predict the 1/0 infection status of animals in the field. We interacted the variable species (the species from which the individual sample was collected) with all fixed-effect predictor variables to clarify the factors driving infection patterns in each of the different species included in our analyses. We started with a global model that included all interaction terms, all biotic and abiotic predictor variables, and the wetland site random effect. We also ran a second intercept only model. We then simplified the global model by first removing each interaction term in a factorial design to determine if a simplified model (with fewer interaction terms) provided a better fit for the data. We selected among these models using the Akaike Information Criterion (AIC). The model with the lowest AIC was considered the best-supported model by the data. Models with a ΔAIC >2 than the best-supported model were considered not well supported by the data [39].

The best-supported GLMM model predicting infection in individuals included the species x *A. woodhousii* density, species x *P. triseriata* density and species x *L. catesbeianus* density interaction terms, indicating that density of species at a wetland can have differential impacts on the likelihood of infection in other species (Table 1). To clarify the role that the density different species play in influencing infection in other species, we completed a follow-up GLMM analysis using the density of *A. tigrinum*, *A. woodhousii*, *P. triseriata*, and *L. catesbeianus* as fixed-effect predictor variables predicting the 1/0 infection in *A. tigrinum*, *A. woodhousii*, *P. triseriata* and *L. catesbeianus*, respectively, with wetland site as a random effect. Due to the small number of sites where *A. woodhousii* and *P. triseriata* were found sympatric, we were unable to use density of *A. tigrinum* as a predictor of infection in *A. woodhousii* (and vice versa). We also used a correlation test to determine if the density of species at a wetland was statistically significantly related to the species richness at a wetland.

**Table 1:** Results from best-supported GLMM models predicting 1/0 Bd infection in individuals and quantitative Bd infection load.

Outcome Variable	Predictor Variable	Coefficient	Standard Error	P-value^b^
Infection (1/0)^a^	Intercept	−19.900	8.070	**0.01**
	Density of ANWO^c^	0.002	0.002	0.35
	Density of PSTR^d^	0.000	0.001	0.55
	Density of LICA^e^	0.039	0.016	**0.01**
	Species (AMTI^f^)	1.850	2.230	0.41
	Species (ANWO)	4.160	2.450	0.09
	Species (LICA)	7.830	2.250	0.00052
	Species richness	−1.110	0.492	**0.02**
	Hydroperiod (temporary)	1.350	1.030	0.19
	Wetland area	−0.801	0.457	0.08
	Wetland elevation	0.008	0.004	**0.04**
	ANWO Density*Species (AMTI)	−0.006	0.003	0.05
	ANWO Density*Species (ANWO)	−0.002	0.002	0.32
	ANWO Density*Species (LICA)	0.000	0.002	0.87
	PSTR Density*Species (AMTI)	0.003	0.001	0.063
	PSTR Density*Species (ANWO)	−0.309	94.100	0.99
	PSTR Density*Species (LICA)	0.001	0.019	0.95
	LICA Density*Species (AMTI)	0.019	0.043	0.66
	LICA.Density*Species (ANWO)	−0.013	0.018	0.46
	LICA Density*Species (LICA)	−0.037	0.016	**0.02**
Quantitative Load	Intercept	−1.487	1.247	0.23
	Total amphibian density	−0.001	0.001	0.43
	Species (AMTI)	−4.119	1.706	**0.02**
	Species (ANWO)	0.909	1.267	0.47
	Species (LICA)	3.996	1.154	**0.00053**
	Total density*Species (AMTI)	0.003	0.001	0.02
	Total density*Species (ANWO)	−0.003	0.001	**0.04**
	Total density*Species (LICA)	0.002	0.001	0.11

a Individuals with detected Bd infection = 1, individuals without detected Bd infection = 0.

b Statistically significant p-values (p<0.05) in bold.

c ANWO is *Anaxyrus woodhousii*;

d PSTR is *Pseudacris triseriata*;

e LICA is *Lithobates catesbeianus*;

f AMTI is *Ambystoma tigrinum.*

We then repeated the GLMM analyses to determine the suite of abiotic and biotic factors that best predict the quantitative Bd infection load of swabbed individuals encountered in the field study using a mixed effects model with a negative binomial distribution and site as random effect. We used a negative binomial distribution because the quantitative Bd load data are highly over-dispersed [40]. The quantitative load dataset also includes a larger number of zeros than would be expected in a negative binomial distribution [40]. To account for this we fit all models first with a negative binomial error distribution and again with a zero-inflated negative binomial error distribution. In all cases, the zero-inflated model produced an AIC value much smaller (ΔAIC >50 for all models) than the model without zero-inflation, indicating that a zero-inflated negative binomial is a considerably better error distribution for these data [40].

We created three candidate models of fixed-effects to predict the quantitative Bd load from individuals sampled in the field. The first model was fit with biotic predictor variables, the second with abiotic predictor variables and the last was an intercept only model. All three of these models included wetland site as a random effect. A model including both the biotic + abiotic category of variables was over-specified and thus was not run. In the biotic category of predictor variables we included: the number of species at a wetland as well as the total density of all species at a wetland (calculating by summing the density for each individual species at a wetland). We used total species density as a predictor rather than the species-level densities due to non-convergence of the model in which density for each species at a wetland was included as a separate predictor variable. The abiotic predictor variables included in the models to predict quantitative Bd infection load were: area, hydro period and elevation of a wetland. We interacted species with all predictor variables to determine which variables are most important for driving infection patterns in each species sampled in the field study. We compared among all models using AIC, with models with a ΔAIC >2 than the best-supported model considered not well supported by the data [39].

### Statistical analyses of laboratory infection data

During our laboratory infection experiment, several individuals were not infected with Bd, even after two 24-hour exposures to either ∼10,000 or ∼200,000 zoospores (Table S1 in File S1). We used a binomial GLM to determine if species, treatment group (low treatment or high treatment) or an interaction between species and treatment group was a significant predictor of whether or not an individual was infected with Bd.

For those individuals that were infected with Bd, we determined the magnitude of their zoospore output over the 62-day time course of the experiment using Simpson’s numeric integration. This allowed us to calculate the area under the curve of zoospore output over time for each individual. We log transformed the integrated area under the curve for each individual and fit a linear model with the variables: species, treatment group (high or low infection) and a species x treatment group interaction. The residuals of this analysis were normally distributed, suggesting that the log transformation was sufficient to normalize the error [40]. To account for the difference in sizes among the species we utilized in the laboratory experiment, we translated the weight of each individual included in the experiment into a surface area according to [41], [42]. To provide a measure of zoospore output/cm^2^, we divided the zoospore output of each individual at each soak date by the individual’s surface area. We then re-calculated the area under these curves using Simpson’s numeric integration and fit a second linear model with the variables species, treatment group (high or low infection) and a species x treatment group interaction predicting the surface area standardized zoospore output as the dependent variable.

To determine if the number of zoospores produced by each species at a wetland has a relationship with the probability of infection or the presence or absence of sympatric species, we paired data from the laboratory experiment and from the field survey to calculate an estimate of the force of infection of each species. We multiplied the average zoospore output over time produced by a given species in the laboratory experiment by the proportion of infected individuals and the density of that species at a field site to determine the number of zoospores produced by each species at a given wetland, a measure that provides a proxy of the ‘force of infection’ of each species at each wetland. We used this calculated force of infection value of each species as predictor variables to predict the proportion of infected individuals of each species at a wetland given using four binomial generalized linear models (GLM). We used a binomial GLM model because our outcome variable was in the form of a proportion (number of infected individuals/total number of individuals swabbed). However, the binomial models predicting the proportion of infected individuals of each species suggested over-dispersion (residual deviance/degrees of freedom >1) [40], so we re-fit these models with a quasibinomail distribution. Finally, we summed the force of infection of all species present at each wetland, and used this to predict the presence or absence of each species at that wetland (*A. tigrinum*, *A. woodhousii*, *P. triseriata, L. catesbeianus*) using generalized linear models with a binomial error distribution.

## Results

### Field survey

During our field survey that occurred during the months of June – August 2011 we detected at least one amphibian at 52 of the 63 haphazardly selected wetlands. See Table 2 and Table S3 in File S1 for specific information about the number of infected sites, the number of individuals of each of the different species encountered, and the number of infected individuals across wetland community types. We used generalized linear mixed effects modeling (GLMM) to determine the suite of abiotic and biotic variables that relates to the probability of infection in the species encountered in our field survey using the glmmADMB package in R [38]. We found that a single model was best-supported by the data (ranked by AIC) [39]. This final model included both biotic and abiotic fixed-effect predictor variables and wetland site as a random effect (Table S2 in File S1). To determine what drives infection patterns in populations of each species sampled in the field survey, we interacted the variable species (a categorical variable indicating which species a sample was collected from) with all abiotic and biotic predictor variables. The glmmADMB package uses “dummy” or “treatment” coding for categorical variables [38], so we manually set the reference condition for the “species” variable to be *P. triseriata,* as this is the species that showed the overall lowest infection across sites and thus serves as our baseline of Bd infection. All coefficients provided for both the single “species” variable, as well as all interaction terms with the “species” variable (see Table 1) represent the difference of each class (either species *A. woodhousii*, *A. tigrinum*, or *L. catesbeianus)* to the *P. triseriata* reference class, thus the effect of each species is only in relation to this baseline level [38].

**Table 2:** Prevalence of Bd infection across amphibian communities and populations.

	Wetland community type		
	LICA only^a^	Co-occurring^a^	Native only^a^
# Wetlands sampled	11	14	11
# Wetlands Bd+^b^	11	10	7
Site level prevalence^b^	100%	71.40%	63.60%
# *L. catesbeianus* sampled	270	154	-
# *L. catesbeianus* Bd+	167	47	-
*L. catesbeianus* prevalence^c^	62.50%	30.50%	-
# *A. tigrinum* sampled	-	59	106
# *A. tigrinum* Bd+	-	5	29
*A. tigrinum* prevalence^c^	-	8.50%	27.40%
# *A. woodhousii* sampled	-	191	79
# *A. woodhousii* Bd+	-	9	2
*A. woodhousii* prevalence^c^	-	4.70%	2.50%
# *P. triseriata* sampled	-	125	163
# *P. triseriata* Bd+	-	3	4
*P*. *triseriata* prevalence^c^	-	2.40%	2.50%

a LICA only are wetlands where we detected only *L. catesbeianus* populations; co-occurring wetlands are those where we detected the presence of both *L. catesbeianus* populations and at least one other native amphibian population, native only are wetlands where we detected populations of at least one native amphibian species but no *L. catesbeianus* individuals.

b Wetlands were designated Bd+ if at least one sample collected from the wetland tested positive for Bd. The site level prevalence is the proportion of sampled wetlands of each wetland community type with at least one individual that tested positive for Bd.

c The species level prevalence is the proportion of sampled individuals of each species that tested positive for the pathogen Bd at each of the different wetland community types.

Prevalence of Bd infection determined from all amphibian populations at the 36 wetlands in the three different wetland community types from which we collected population level Bd data. The North American bullfrog (*L. catesbeianus*) was the only non-native amphibian species encountered, while tiger salamanders (*A. tigrinum*), Woodhouse’s toads (*A. woodhousii*), and western chorus frogs (*P. triseriata*) are all native amphibian species in the Colorado study system. We were able to obtain population-level Bd estimates data from only one population of Northern leopard frogs (*L. pipiens*), and therefore we did not include this species into our analysis. See Table S3 in File S1 for further detail about Bd infection in *L. pipiens* in our study.

The best-supported model predicting infection (1/0- infected/not infected) included all of the abiotic wetland characteristics (area, elevation and wetland hydro period), though only elevation was statistically significantly related to Bd infection, with higher elevation wetlands having a higher probability of Bd infected individuals (see Table 1 for all associated p-values, coefficients and standard errors). For reference, the range of elevations included in this dataset are 1503–2087 meters above sea level, representing the variation in topography in the high plains Front Range region, east of (and not including) the Rocky Mountains. The best supported model included five out of the six biotic predictor variables including species, species richness, and density of *A. woodhousii,* density of *P. triseriata*, and density of *L. catesbeianus* (Table 1). The density of *A. tigrinum* was not included in the best-supported model predicting (1/0) Bd infection (Table S2 in File S1). When considering the “species” variable alone, we found that the probability of a sampled individual testing positive for Bd was statistically significantly more likely if that individual was of the species *L. catesbeianus* compared to *P. triseriata* (Table 1). The final model included the density of *P. triseriata*, *L. catesbeianus* and *A. woodhousii* as predictors, though only the density of *L. catesbeianus* was statistically significant, with wetlands supporting more dense populations of *L. catesbeianus* more likely to supported Bd infected individuals (Table 1). Finally, there was an increased likelihood of detecting Bd at wetlands with overall lower species richness (Table 1).

**Figure 2:**
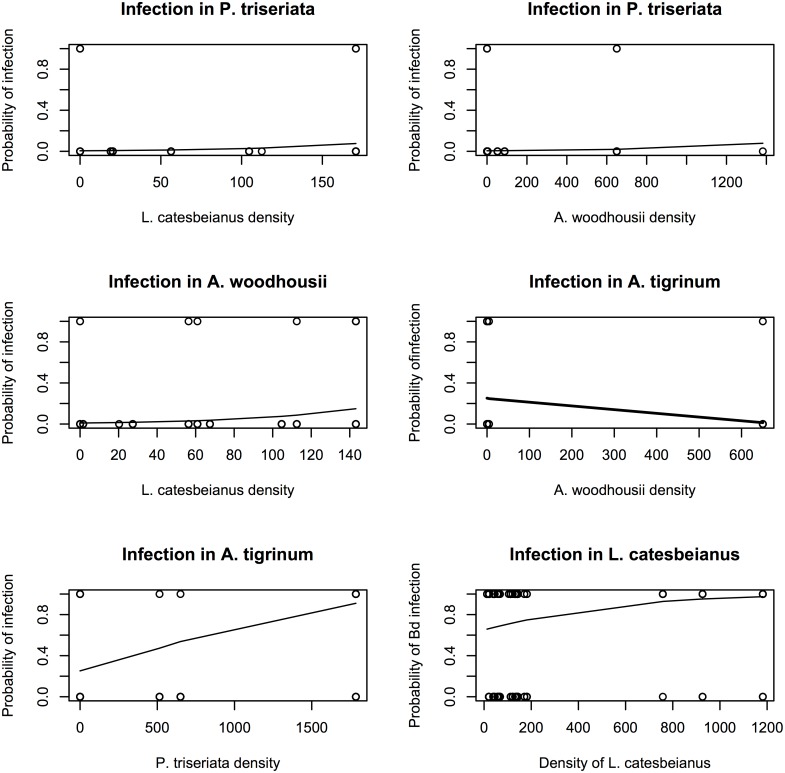
Relationship between density of host species and the probability of Bd infection in individuals. The density of *L. catesbeianus* at a wetland was a statistically significant predictor of infection in both *A. woodhousii* (p = 0.0065) and *P. triseriata* (p = 0.024), as obtained from generalized linear mixed effects models predicting the probability of Bd infection in individual species encountered in the field survey. The density of *A. tigrinum* (not shown), *A. woodhousii*, and *P. triseriata* were all statistically significant predictors of infection in *A. tigrinum* (p = 0.034, 0.006, 1.5e-5, respectively). The density of *L. catesbeianus* at a wetland was a nearly statistically significant predictor of infection in that species (p = 0.093), though the density of no other amphibian species was statistically significantly related to infection in *L. catesbeianus* (p>0.3 for all other species).

We created four generalized mixed effects models to clarify the relationship between the density of each species and the probability of infection in other species at that wetland, again using sites as a random effect. The density of *L. catesbeianus* at a wetland was a statistically significant predictor of infection in both *A. woodhousii* (p = 0.0065) and *P. triseriata* (0.024), though the magnitude of the effect was small for both species (*A. woodhousii* coefficient = 0.002*; P. triseriata* coefficient = 0.016, Figure 2). The density of *A. tigrinum*, *A. woodhousii*, and *P. triseriata* were all statistically significant predictors of infection in *A. tigrinum (*p = 0.034, 0.006, 1.5e-5, respectively, Figure 2). The density of *L. catesbeianus* at a wetland was a nearly statistically significant predictor of infection in that species (p = 0.093), though the density of no other amphibian species was statistically significantly related to infection in *L. catesbeianus* (p>0.3 for all other species) (Figure 2). We completed a correlation analysis to determine the relationship between species richness and amphibian density, and found a slightly negative association between species richness and the total density of amphibians at each wetland (coefficient = −0.05, 95% CI: −0.38, 0.28, d.f. = 34), though this relationship was not statistically significant (p = 0.73).

Next, to predict the quantitative Bd infection load in amphibian individuals encountered in our field survey, we used generalized linear mixed effects modeling with a negative binomial error distribution and a suite of abiotic and biotic predictor variables (Table S2 in File S1). We created models using both the standard negative binomial error distribution as well as the zero inflated negative binomial distribution, and found that the zero-inflated negative binomial models produced models that were better supported by our data (ΔAIC >50). We created a suite of models (see Table S2 in File S1) and ranked these models according to their AIC. The model with the lowest AIC was considered the best-supported model by the data [39]. Any model within 2 AIC of the best-supported model was also considered well supported by the data [39]. In our analysis, however, we identified only a single best-supported model. The best-supported model predicting infection load included only the biotic categories of variables as fixed effects and wetland site as a random effect. As with the GLMM models we used to predict 1/0 infection in individuals, we manually set the reference condition for the “species” variable to be *P. triseriata*, because this species had the lowest Bd infection loads and serves as a baseline of Bd infection levels. The results of this analysis were similar to the binomial GLMM we used to predict 1/0 infection in individuals, however unlike the binomial GLMM, species richness at a site was not included in the best-supported model. *L. catesbeianus* individuals were statistically significantly more likely to have higher infection loads than any other species (Table 1). Additionally, the total density of all amphibians at a site was significantly and negatively related to infection load in *A. woodhousii* individuals and positively related to infection load *in A. tigrinum,* though again this effect is only in comparison to the effect of total density on infection load in *P. triseriata* individuals.

### Laboratory infection study

**Figure 3:**
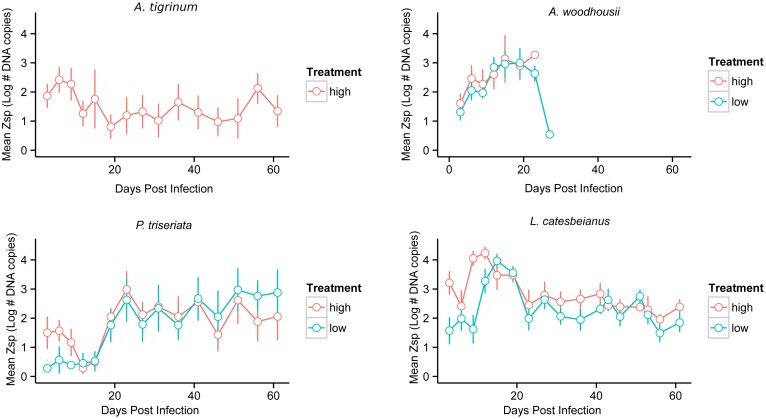
Plots of the mean zoospore output over time for four species experimentally infected with Bd. Mean zoospore output (with standard error bars) of individuals within each treatment group (high and low) over the 62-day time course of the laboratory experiment. Zoospore output over time calculated by taking Simpson’s numeric integral to find area under curve of zoospore output over time for each species. Treatment group (high or low treatment) alone did not have a statistically significant relationship with the integrated zoospore output of individuals over time (p = 0.62) in a linear mixed model predicting total zoospore output over time. No species x treatment group interactions were significantly related to total zoospore output for any species (p>0.67 for all species x treatment group interactions). The species *A. tigrinum* and *A. woodhousii* had a statistically significant negative association with zoospore output in comparison to the total zoospore output from *L. catesbeianus* individuals (p* = *0.029, 0.023, respectively).

We used a generalized linear model (GLM) with a binomial error distribution to determine if treatment group (high or low), species, or an interaction between treatment and species were statistically significantly associated with the likelihood of an individual becoming infected in the laboratory experiment. We found no relationship with either species or treatment group (low or high infection) (p = >0.9 for all species, treatments, and species x treatment interactions). A second linear mixed model was used to determine if treatment group or species was related to the total integrated zoospore output produced by each individual in the laboratory experiment. We set *L. catesbeianus* as the reference group for the categorical predictor variable “species”. We did not find that treatment group (high or low treatment) alone had a statistically significant relationship with the integrated zoospore output of individuals over time (p = 0.62). Also none of the species x treatment group interactions were significantly related to total zoospore output for any species (p>0.67 for all species x treatment group interactions). We did find that all other species (*A. tigrinum, A. woodhousii*, and *P. triseriata*) had a negative association with zoospore output in comparison to the total zoospore output of *L. catesbeianus.* This relationship was statistically significant for *A. tigrinum* (p = 0.028) and *A. woodhousii* (p = 0.023) and nearly statistically significant for *P. triseriata* (p = 0.062) (Figure 3). To account for the difference in body size among the species in the laboratory experiment, we divided the zoospore output of each individual by the individual’s surface area to provide a measure of zoospore output/cm^2^. After accounting for the difference in size among the species utilized in our laboratory experiment only *A. woodhousii* was statistically significantly negatively related to the total number to zoospores released over the time course of the experiment relative to the number of zoospores produced by *L. catesbeianus* individuals (p = 0.0312, coefficient: −1.1 s.e. = 0.50).

We combined data from the field survey and the laboratory infection experiment to determine the force of infection produced by each species at each wetland. We then used this to predict the presence or absence of each species at a wetland using generalized linear models with a binomial distribution. We did not find that the force of infection of any species was a significant predictor of the presence or absence of any other species in the field survey (p>0.5 for all models). We used a second generalized linear model with a quasi-binomial distribution, and found that the force of infection produced by each species at a wetland was also not a statistically significant predictor of infection in any other species (p>0.5 for all models).

## Discussion

Many important pathogens of humans and wildlife infect more than one host species, highlighting the necessity of understanding how these different hosts contribute to pathogen dynamics in the landscape (e.g. [43]). A number of studies have focused on quantifying the differential contribution of host species for zoonotic pathogens (pathogens that infect both human and wildlife species) as such information can have consequences for human health (e.g., [44]). However, there are few examples of such investigations for pathogens that infect exclusively wildlife and even fewer empirical investigations into heterogeneity of hosts for pathogens with transmission that is not mediated by a vector. In this study, we investigated host heterogeneity across a suite of scales and species to clarify how different hosts of the directly transmitted amphibian pathogen Bd respond to and influence pathogen dynamics in the landscape.

Individual, population, and community level variability strongly influence dynamics of many pathogens [7]. In this study, we investigate how host responses at different scales may drive dynamics in a Bd amphibian system. Specifically, we aimed to determine if: 1) populations of different amphibian species in the Colorado Front Range vary in Bd infection across the landscape, 2) amphibian community composition predicts infection (e.g., does the presence or density of any particular species influence infection in sympatric species?), 3) amphibian species vary in their ability to produce infectious zoospores and 4) heterogeneity in host ability observed in the laboratory scales to predict patterns of Bd prevalence in the landscape. This approach allows for a nuanced description of host/pathogen dynamics that may facilitate identifying particular host species that contribute more relative to other species to maintaining or transmitting the Bd pathogen in the landscape.

### Community and population-level patterns of Bd infection

In our large-scale wetland field survey we encountered four native amphibian species (*A. tigrinum, A. woodhousii, L. pipiens*, and *P. triseriata*) and one non-native species (*L. catesbeianus*). We identified *L. catesbeianus* more frequently than any other individual native amphibian species (Table 2, Table S3 in File S1), which has been found in other surveys in this system [28], [29], [30]. Additionally, 100% of the wetlands where we obtained swab samples to test for the pathogen *Batrachochytrium dendrobatidis* from *L. catesbeianus* supported at least one Bd infected individual (Table S3 in File S1). Individuals that tested positive for Bd, as well as individuals with high Bd loads, were statistically significantly more frequently to be of the species *L. catesbeianus* than any native species in this system (Table 1). Additionally, wetlands with increased densities of *L. catesbeianus* were more likely to support Bd infected individuals overall (Table 1), indicating that non-native *L. catesbeianus* may contribute to maintaining the non-native Bd pathogen in the Colorado Front Range landscape.

We used GLMM analyses to determine the relationship between density of amphibian populations and Bd infection in other host species. We found that the density of *L. catesbeianus* at a wetland was a statistically significant predictor of infection in both *P. triseriata* and *A. woodhousii*, with increased densities of *L. catesbeianus* increasing the probability of detecting a Bd infected individual of each of these species (Figure 2). This indicates that *L. catesbeianus* populations may play a role in influencing infection dynamics in sympatric native species. However, we also identified Bd infection in a number of native amphibian populations allopatric with *L. catesbeianus* populations (Table 2, Table S3 in File S1). For example, we detected Bd in nearly 60% of *A. tigrinum* populations found allopatric with *L. catesbeianus* and in nearly 40% of *P. triseriata* populations allopatric with *L. catesbeianus* (Table S3 in File S1). Additionally, there was not a relationship with infection in *A. tigrinum* or the density of *L. catesbeianus* at a wetland (Figure 2). Together, these results highlight that while *L. catesbeianus* populations are widespread and broadly infected with Bd across the landscape, Bd infection in native amphibian populations does occur independent of the presence of *L. catesbeianus* and that multiple hosts in this system are likely important for driving patterns of Bd infection in the landscape.

When further considering predictors of Bd infection at the landscape level, we found that the number of species at a wetland was statistically significantly negatively related to the probability of detecting Bd infected individuals at that wetland. This trend is consistent with a broad definition of a dilution effect, which refers to the condition that occurs when high host diversity (in this case measured as amphibian species richness) is associated with lower disease risk [46]. Other studies have identified a potential dilution effect in Bd systems in a laboratory setting [47], though it is unclear if the relationship observed in this study is indicative of a true dilution effect. Density of some species at a wetland (primarily *L. catesbeianus*) is related to Bd infection in our system (Figure 2). While we did not find that the total density of species was statistically significantly negatively related to the species richness at a site, it is still possible that density-richness relationships may play a role in driving the observed pattern between species richness and infection in our systems. Additionally, unmeasured site level characteristics, such as microorganisms that can act as Bd zoospore predators [48], could co-vary with species richness in our system and could also be driving the observed relationship between richness and Bd infection. Further work needs to be done to clarify what role, if any, increased species richness may play in reducing Bd infection risk in amphibian populations at the landscape scale.

Lastly, we also found that Bd infection in individuals is related to abiotic factors, with the best-supported model predicting Bd infection including the abiotic characteristics area, wetland hydro period, and elevation (Table 1). The effect of these predictors appears to be relatively consistent across species, as no abiotic x species interaction terms were included in the best-supported model (Table 1). Elevation was the only statistically significant predictor variable included in the GLMM model predicting Bd infection, with higher elevation wetlands more likely to support Bd infected individuals (Table 1). Many of the documented Bd driven-declines in amphibian populations globally have occurred in high elevation amphibian populations, including in boreal toads in Colorado, which are historically distributed above 2100 m (or about 7,000 feet) elevation [25], [26], [49], [50]. We conducted this study within the known elevation range of *L. catesbeianus* in this system (1503–2087 m), which is lower than the range of boreal toads that have undergone known Bd-driven declines in Colorado. However, it appears that in our system even fine scale increases in elevation may relate to Bd infection in amphibians.

### Individual and species-level patterns of Bd infection

**Figure 4:**
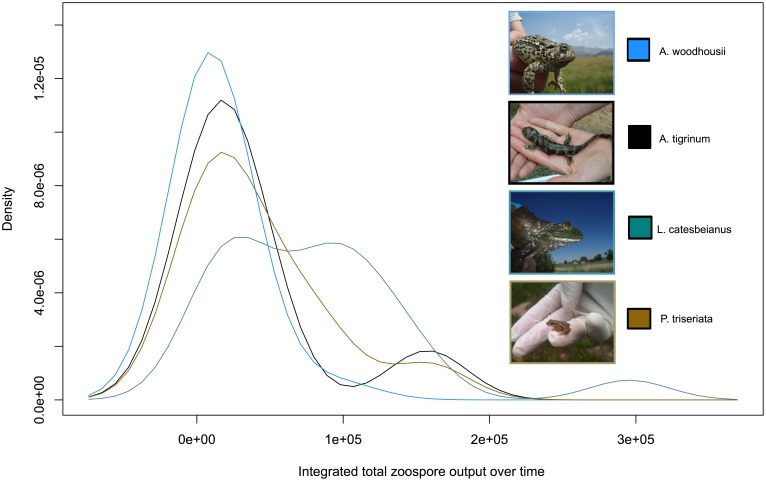
Kernal density plot representing the distribution of the integrated zoospore output over time for individuals. Species included in the laboratory infection: *Ambystoma tigrinum* individuals (n = 8), *Anaxyrus woodhousii* individuals (n = 25), *Pseudacris triseriata* individuals (n = 12) and *Lithobates catesbeianus* individuals (n = 21). Total zoospore output over time calculated by taking Simpson’s numeric integral to find area under curve of zoospore output over time for each individual.

Clarifying potential heterogeneity among species in their ability to act as hosts for Bd (i.e. their ability to become infected with the pathogen and once infected produce the infective zoospore stage of the pathogen) can lend important insight into the amphibian/Bd system as a whole. If some species produce larger amounts of the infective zoospore relative to other species, this could have important implications for transmission dynamics of this pathogen in the environment. Currently, to our knowledge, no studies have been done to investigate the differences among amphibian species in their output of the infectious Bd zoospore stage over time. We conducted a laboratory infection experiment, in which we infected individuals of four different species with the same locally isolated Bd-GPL strain of the pathogen and compared zoospore output across the individuals and species over time. We found no effect of treatment dose (high versus low infection) in the total number of zoospores produced by any of the species over time. We did find that *A. woodhousii* and *P. triseriata* were statistically negatively related to the total zoospore output relative to the total zoospore output of *L. catesbeianus* individuals (*A. woodhousii:* p = 0.29, coefficient = −1.93; *P. triseriata*: p = 0.023, coefficient = −1.96, Figure 3). After accounting for differences in body size among the species included in the laboratory study, only *A. woodhousii* was statistically significantly related to Bd zoospore output, with a negative output relative to the total zoospores produced by *L. catesbeianus* individuals (p = 0.031, coefficient = −1.0). Kernal density plots of the zoospore output of individuals of each species included in our laboratory study demonstrate that there is large variability among individuals in their zoospore output over time (Figure 4). For all species, one or two individuals tended to produce more of the zoospore stage relative to other individuals of the same species- though this trend was less pronounced for *L. catesbeianus* individuals (Figure 4). Rather, *L. catesbeianus* individuals tended to more consistently produce a higher number of Bd zoospores relative to the other species included in the laboratory study (Figure 4).

The general trend for a few individuals to contribute relatively largely to the pathogen pool is consistent with patterns seen in many other pathogens (e.g. [51]). The intraspecific variability in ability to produce infective zoospores could be driven by a number of different factors. One possibility is that this variability is a result of previous unknown exposure of the Bd pathogen. All individuals (early metamorphic stages) included in our laboratory experiment were collected from the field. Individuals of a given species were collected from multiple wetlands, and we targeted collecting individuals from wetlands where little or no Bd infection had been detected in the field survey. We also screened all individuals for Bd infection prior to inclusion in the experiment, and included only uninfected individuals in our study. However, we still cannot rule out that some species could have been infected with Bd and lost infection before capture and inclusion in our laboratory study, though this is unlikely given that we used early life stages of amphibians. Other studies have found little evidence to suggest that amphibians may mount an adaptive immune response to Bd infection (e.g. [52], [53]), suggesting that even if such infections had occurred it may not have played a role in influencing these individuals’ response to Bd infection in our laboratory study. Regardless of the drivers of this variability, it does appear that some individuals may be more likely to contribute more to Bd pathogen dynamics relative to other individuals of the same species. Future studies should consider individual-level heterogeneity, even among individuals at the same life stage, as a potentially important factor driving Bd pathogen dynamics (e.g., [54]).

Our laboratory study also suggests that there may be potential heterogeneity among species in their ability to acquire infection with Bd. Individuals of the both of the species *A. tigrinum* and *P. triseriata* showed some resistance to Bd infection, and nearly 46% percent of *A. tigrinum* individuals and 25% of *P. triseriata* individuals did not obtain Bd infection, even after being exposed to 2 doses of ∼10,000 or ∼200,000 zoospores. Neither species nor treatment group were statistically significant predictors of the likelihood of an individual becoming infected in the laboratory experiment (p>0.99 for all species and treatments). However, our laboratory experiment had relatively low samples sizes and with greater individuals more clarity could be gained as to whether *A. tigrinum or P. triseriata* demonstrate real resistance to Bd infection.

### Conclusions across scales

Data from the field survey indicate that *L. catesbeianus* populations are broadly infected across the landscape (Table 2), and the density of this species at a wetland is related to an increased probability of infection in some other native species (Figure 2). By investigating dynamics across multiple scales, we are better able to disentangle possible mechanisms that may be driving this pattern. The high Bd infection in *L. catesbeianus* populations may be in part explained by niche overlap between *L. catesbeianus* and Bd, which has been shown to enhance transmission of Bd in other systems [45]. Relative to the native amphibians included in the field study, *L. catesbeianus* are more aquatic, have a much longer aquatic tadpole stage [18] and also tend to occur most frequently in permanent wetlands than in temporary wetlands [28]. The Bd pathogen is aquatic and cannot withstand desiccation [9], thus the species *L. catesbeianus* may have higher infection levels relative to native species due to their greater aquatic life-history characteristics relative to the other species in this system. The highly aquatic life history strategy of *L. catesbeianus* relative to other native species, combined with their tendency to produce more of the infectious zoospore stage of the Bd pathogen compared to some native species, may increase the likelihood of this species becoming infected with Bd in the landscape and could explain the higher prevalence and Bd infection load observed in populations of this species in the field.

Furthermore, of the species included in our laboratory study, *L. catesbeianus* individuals are on average 10 times larger in size than the native Colorado species of the same age class. Given that Bd infects the keratinized skin of amphibians, we predicted that the larger body size of *L. catesbeianus* may relate to the ability of this species to produce more of the infective Bd zoospore stage, as the pathogen could have more area to spread and colonize. We found that *L. catesbeianus* did produce more of the infectious Bd stage relative to both *P. triseriata* and *A. woodhousii* individuals. Though after accounting for the difference in body size, *L. catesbeianus* showed only and increased level of zoospore output relative to *A. woodhousii*. None of the *A. woodhousii* individuals survived the full 62-days of the infection experiment, and thus their total zoospore output was truncated relative to all other species included in the laboratory experiment. Overall, this result suggests the larger body size of *L. catesbeianus* individuals could be another potential mechanism to explain the increased Bd load or prevalence of Bd infection found in individuals of this species in the landscape.

When looking across organismal life-history strategies, ‘fast-lived’ species (e.g. faster lived species with shorter life spans and greater reproductive rates) are hypothesized to be the most likely species to act as biotic disease reservoirs (e.g. [55]). Invasive species are often fast-lived species, and thus established invasive species are hypothesized to generally acts as more competent disease reservoirs as similar life-history traits that make a species a good invader may also relate to reservoir potential (e.g. [55]). This phenomenon has been well-documented in some plant systems (e.g. [56]), though is less well-documented in other systems. The North American bullfrog was the only non-native species encountered in our field study. Interestingly, relative to native species in the Colorado Front Range system, *L. catesbeianus* have a much larger body size and show a protracted larval development, and both of these characteristics of the invasive *L. catesbeianus* appear to relate to the potential increased prevalence of Bd infection in this species relative to the native species encountered. This highlights how important context-specific dynamics of host/pathogen systems are for driving landscape-level infection dynamics in some systems.

The observed increased probability of infection in populations of some native species at wetlands where *L. catesbeianus* are more dense (Figure 2) may be due either to direct transmission of Bd from *L. catesbeianus* to native species or through density-dependent effects. To better clarify if *L. catesbeianus*, or any other native amphibian species, are driving infection patterns in other species by increasing the number of zoospores present in a wetland, we paired landscape-level patterns of occurrence and Bd prevalence data with data obtained in the laboratory study to calculate an estimate of the average force of infection of each species (an estimate of the number of Bd zoospores produced by populations of each species at a wetland). We then used this measure to predict presence/absence of other species across the landscape, as well as Bd infection on other species. We did not find that the average force of infection of any species, including *L. catesbeianus,* was statistically significantly related to either Bd infection or the presence or absence of any species in the field survey. Thus, it appears that *L. catesbeianus* may influence dynamics of the pathogen Bd in wetlands by increasing the overall density of hosts above some threshold, allowing for greater infection across all species present in these wetlands. This may be a better explanation of the observed relationship between infection in native species and *L. catesbeianus* density (Figure 2), than an alternative explanation that assumes direct transmission of the Bd pathogen from *L. catesbeianus* to native species.

We found that the density of *A. tigrinum* at a wetland was not included in our best-supported GLMM model predicting overall Bd infection across the landscape (Table 1). This could in part be due to a vestige of the density estimates collected in the field. Of the species included in the field survey, *A. tigrinum* can be the most difficult to detect due to their lack of vocalization and cryptic nature. We rarely detected *A. tigrinum* in the visual encounter survey and density estimates for this species were primarily based on seine and sweep-net data. It is possible that due to the difficult of detecting *A. tigrinum* relative to the other species included in this study, we underestimated densities of this species at some wetlands. If our *A. tigrinum* density estimates were artificially low, this could have obscured any potential relationship between *A. tigrinum* densities and Bd infection dynamics in wild populations.

The level of Bd infection in a wetland may also truly be independent of the density of *A. tigrinum* populations. The potential resistance to infection of *A. tigrinum* and *P. triseriata* individuals seen in our laboratory experiment could partially explain the lower prevalence (compared to *L. catesbeianus*) of Bd infection in these populations in the field (Table 2, Table S3 in File S1). However, it is important to note that of the native species from which we collected population level estimates of Bd infection, *A. tigrinum* had the highest prevalence of Bd infection (21% of all sampled individuals infected) (Table 2, Table S3 in File S1). The relatively high prevalence of Bd infection in some populations of *A. tigrinum* (for example at one wetland 23 out of 32 swabbed (∼72%) *A. tigrinum* individuals were Bd positive (Table S3 in File S2)) suggests that even if on the small scale some individuals show some resistance to infection, at the larger scale populations of *A. tigrinum* are still capable of acquiring high levels of Bd infection in the field. This result again indicates that individual-level trends observed on the small scale do not always directly correspond to landscape level patterns of infection, highlighting the importance of investigating infection dynamics across scales.

While it is well established that *L. catesbeianus* have played an important role in transporting Bd on a global scale [19], [20], [21], [22], [23], few studies have investigated how this species influences pathogen dynamics once both the pathogen Bd and the *L. catesbeianus* host have become established in a non-native landscape. In our system, we did not find clear links between Bd infection in *L. catesbeianus* and Bd infection in native species, though we did find that this species is responsible for the majority of the total distribution of the Bd pathogen in the landscape and some evidence that this species may influence Bd dynamics in other species; likely through increasing overall host densities at some wetlands. However, we also found that native amphibian species, especially *A. tigrinum*, showed a relatively high prevalence of Bd infection at wetlands allopatric of *L. catesbeianus*. Suggesting that multiple hosts are important for maintaining the Bd pathogen in this system. At the landscape scale, we observed large variation in infection patterns across different host populations and communities (Table 2). At the individual level we also found a great deal of intraspecific variation (Figure 4). The large degree of heterogeneity across scales suggests that population or community level characteristics such as host densities, as well as landscape characteristics such wetland elevation, are likely equally important for driving patterns of Bd infection in the Colorado system than is the presence or absence of a single species in the landscape. Few studies have directly addressed the responses of multiple host species at different scales to infection with a directly transmitted pathogen. Our results highlight that species composition, abiotic factors, and individual characteristics of host species and the pathogen are all important drivers of pathogen dynamics in wild host/pathogen systems.

## Supporting Information

File S1 **Supporting tables.** Table S1, Number (n) of individuals included in the laboratory infection experiment. The starting (n) is the number of individuals of each species that were infected with Bd at the beginning of the experiment. The mortality loss is the number of individuals that died before the beginning of experiment (within the first 3 days post-infection). The number of resistant individuals are those that were infected with either ∼10,000 (low treatment) or ∼200,000 (high treatment) Bd zoospores two times, but did not test positive for Bd either 3 or 6 days post infection. The final (n) is the number of individuals for each species and treatment group from which we obtained data used in our analyses. AMTI is *Ambystoma tigrinum*; ANWO is *Anaxyrus woodhousi*i, PSTR is *Pseudacris triseriata*; LICA is *Lithobates catesbeianus*. Table S2, All models used in model selection to determine the best-supported model predicting 1/0 infection in individuals and quantitative Bd infection load. Predictor variables were either included (1), or not included (0) in each model. Models were ranked according to their AIC (Akaike Information Criterion). The ΔAIC is the difference between each model and the best-supported model. All models with a ΔAIC <2 are considered well supported by the data. Only one best-supported model was identified to predicting both 1/0 Bd infection and quantitative Bd infection load. K is the number of parameters included in each model. AMTI is *Ambystoma tigrinum*, ANWO is *Anaxyrus woodhousii*, LICA is *Lithobates catesbeianus*, and PSTR is *Pseudacris triseriata*. Table S3, Landscape-level occurrence and site-specific Bd infection data for the five amphibian species encountered in our field study. ^a^The number of wetlands where each species was detected in our survey of landscape –level amphibian occurrence. ^b^The proportion of those wetlands included in our survey of landscape-level amphibian occurrence (n = 52), which supported each amphibian species. ^c^Indicates whether or not a species was observed at a given wetland. 1 = species observed, 0 = species not observed. ^d^We detected at least one individuals of the species *L. pipiens* at 3 wetlands, though only one supported a large enough population for us to gather a population-level estimate of Bd infection prevalence. Due to the small number of *L. pipiens* encountered in our field survey, we did not include this species in any analyses investigating patterns of Bd infection across the landscape.
(DOCX)
