# Supplementary material for: Investigating Differences across Host Species and Scales to Explain the Distribution of the Amphibian Pathogen Batrachochytrium dendrobatidis
Source: PLoS One. 2014 Sep 15;9(9):e107441. doi: 10.1371/journal.pone.0107441 (PMC4164663; doi:10.1371/journal.pone.0107441)
Supplement: File S1 — Supporting tables. Table S1, Number (n) of individuals included in the laboratory infection experiment. The starting (n) is the number of individuals of each species that were infected with Bd at the beginning of the experiment. The mortality loss is the number of individuals that died before the beginning of experiment (within the first 3 days post-infection). The number of resistant individuals are those that were infected with either ∼10,000 (low treatment) or ∼200,000 (high treatment) Bd zoospores two times, but did not test positive for Bd either 3 or 6 days post infection. The final (n) is the number of individuals for each species and treatment group from which we obtained data used in our analyses. AMTI is Ambystoma tigrinum; ANWO is Anaxyrus woodhousii, PSTR is Pseudacris triseriata; LICA is Lithobates catesbeianus. Table S2, All models used in model selection to determine the best-supported model predicting 1/0 infection in individuals and quantitative Bd infection load. Predictor variables were either included (1), or not included (0) in each model. Models were ranked according to their AIC (Akaike Information Criterion). The ΔAIC is the difference between each model and the best-supported model. All models with a ΔAIC <2 are considered well supported by the data. Only one best-supported model was identified to predicting both 1/0 Bd infection and quantitative Bd infection load. K is the number of parameters included in each model. AMTI is Ambystoma tigrinum, ANWO is Anaxyrus woodhousii, LICA is Lithobates catesbeianus, and PSTR is Pseudacris triseriata. Table S3, Landscape-level occurrence and site-specific Bd infection data for the five amphibian species encountered in our field study. aThe number of wetlands where each species was detected in our survey of landscape –level amphibian occurrence. bThe proportion of those wetlands included in our survey of landscape-level amphibian occurrence (n = 52), which supported each amphibian species. cIndicates wh [file pone.0107441.s001.docx]

| **Species** | **AMTI** | | **ANWO** | | **PSTR** | | **LICA** | |
| --- | --- | --- | --- | --- | --- | --- | --- | --- |
| Treatment Group | low | high | low | high | low | high | low | high |
| Starting (n) | 14 | 14 | 14 | 14 | 11 | 10 | 11 | 11 |
| Mortality loss (n) | 9 | 6 | 0 | 3 | 5 | 0 | 0 | 1 |
| Resistant (n) | 5 | 0 | 0 | 0 | 0 | 4 | 0 | 0 |
| Final (n) | 0 | 8 | 14 | 11 | 6 | 6 | 11 | 10 |

**Table S1.**

| **Predictor Variables** | **Models Predicting 1/0 Infection** | | | | | | | | | | | | **Models Predicting Infection Load** | | | | |
| --- | --- | --- | --- | --- | --- | --- | --- | --- | --- | --- | --- | --- | --- | --- | --- | --- | --- |
|  | ***Model Number*** | | | | | | | | | | | | ***Model Number*** | | | | |
|  | ***1*** | ***2*** | ***3*** | ***4*** | ***5*** | ***6*** | ***7*** | ***8*** | ***9*** | ***10*** | ***11*** | ***12*** | ***1*** | ***2*** | ***3*** | ***4*** | ***5*** |
| Area | 1 | 1 | 1 | 1 | 1 | 1 | 0 | 1 | 1 | 1 | 1 | 0 | 0 | 1 | 1 | 1 | 0 |
| Area x Species | 0 | 0 | 0 | 0 | 0 | 0 | 0 | 1 | 0 | 0 | 0 | 0 | 0 | 0 | 1 | 0 | 0 |
| Elevation | 1 | 1 | 1 | 1 | 1 | 1 | 0 | 1 | 1 | 1 | 1 | 0 | 0 | 1 | 1 | 1 | 0 |
| Elevation x Species | 0 | 0 | 0 | 0 | 0 | 0 | 0 | 1 | 0 | 0 | 0 | 0 | 0 | 0 | 1 | 0 | 0 |
| Hydroperiod | 1 | 1 | 1 | 1 | 1 | 1 | 0 | 1 | 1 | 1 | 1 | 0 | 0 | 1 | 1 | 1 | 0 |
| Hydroperiod x Species | 0 | 0 | 0 | 0 | 0 | 0 | 0 | 1 | 0 | 0 | 1 | 0 | 0 | 0 | 1 | 0 | 0 |
| Species | 1 | 1 | 1 | 1 | 1 | 1 | 1 | 1 | 1 | 0 | 1 | 0 | 0 | 0 | 1 | 1 | 0 |
| Species Richness | 1 | 1 | 1 | 0 | 1 | 1 | 1 | 0 | 1 | 0 | 1 | 0 | 0 | 0 | 0 | 0 | 0 |
| Species Richness x Species | 0 | 0 | 0 | 1 | 0 | 0 | 1 | 0 | 0 | 0 | 1 | 0 | 0 | 0 | 0 | 0 | 0 |
| Total Density | 0 | 0 | 0 | 0 | 0 | 0 | 0 | 0 | 0 | 0 | 0 | 0 | 1 | 0 | 1 | 1 | 0 |
| Total Density x Species | 0 | 0 | 0 | 0 | 0 | 0 | 0 | 0 | 0 | 0 | 0 | 0 | 1 | 0 | 1 | 1 | 0 |
| AMTI Density | 0 | 0 | 1 | 1 | 1 | 1 | 1 | 0 | 1 | 0 | 1 | 0 | 0 | 0 | 0 | 0 | 0 |
| AMTI Density x Species | 0 | 0 | 1 | 1 | 1 | 1 | 1 | 0 | 1 | 0 | 1 | 0 | 0 | 0 | 0 | 0 | 0 |
| ANWO Density | 1 | 1 | 1 | 1 | 1 | 1 | 1 | 0 | 0 | 0 | 1 | 0 | 0 | 0 | 0 | 0 | 0 |
| ANWO Density x Species | 1 | 1 | 1 | 1 | 1 | 1 | 1 | 0 | 0 | 0 | 1 | 0 | 0 | 0 | 0 | 0 | 0 |
| LICA Density | 1 | 1 | 1 | 1 | 0 | 1 | 1 | 0 | 1 | 0 | 1 | 0 | 0 | 0 | 0 | 0 | 0 |
| LICA Density x Species | 1 | 1 | 1 | 1 | 0 | 1 | 1 | 0 | 1 | 0 | 1 | 0 | 0 | 0 | 0 | 0 | 0 |
| PSTR Density | 1 | 0 | 1 | 1 | 1 | 0 | 1 | 0 | 1 | 0 | 1 | 0 | 0 | 0 | 0 | 0 | 0 |
| PSTR Density x Species | 1 | 0 | 1 | 1 | 1 | 0 | 1 | 0 | 1 | 0 | 1 | 0 | 0 | 0 | 0 | 0 | 0 |
| Wetland Site | 1 | 1 | 1 | 1 | 1 | 1 | 1 | 1 | 1 | 1 | 1 | 1 | 0 | 1 | 1 | 1 | 1 |
| AIC | 669 | 672 | 674 | 674 | 677 | 677 | 681 | 683 | 683 | 684 | 684 | 742 | 4608 | 4699 | 4706 | 4743 | 4899 |
| Δ AIC | 0 | 3 | 5 | 5 | 8 | 8 | 12.4 | 14 | 14 | 14.9 | 15 | 73 | 0 | 91 | 98 | 135 | 291 |
| K | 12 | 10 | 14 | 14 | 12 | 12 | 12 | 8 | 12 | 4 | 16 | 1 | 2 | 4 | 10 | 7 | 1 |

**Table S2.**

**Table S3.**

| **Large scale wetland survey species occurrence data (number of wetlands = 52)** | | | | | | | | | | | | | | | | | | | | | | | | | |
| --- | --- | --- | --- | --- | --- | --- | --- | --- | --- | --- | --- | --- | --- | --- | --- | --- | --- | --- | --- | --- | --- | --- | --- | --- | --- |
|  | *A. tigrinum* | | | | | | | *A. woodhousii* | | | | | | | *L. catesbeianus* | | | | | *L. pipiens* | | | *P. triseriata* | | |
| # Detected ^a^ |  | | | 5 | | |  |  | | | | 16 | |  |  | | 29 | |  |  | 6 |  |  | 16 |  |
| % Detected ^b^ |  | | | 10% | | |  |  | | | | 31% | |  |  | | 56% | |  |  | 12% |  |  | 31% |  |
| **Site Specific Bd Infection Data (number of wetlands = 36)** | | | | | | | | | | | | | | | | | | | | | | | | | |
|  | | *A. tigrinum* | | | | | | *A. woodhousii* | | | | | | | *L. catesbeianus* | | | | | *L. pipiens* ^d^ | | | *P. triseriata* | | |
| Site | | Obs^c^ | # Swab | | # Infected | | | Obs^c^ | | # Swab | | | # Infected | | Obs^c^ | | | # Swab | # Infected | Obs^c^ | # Swab | # Infected | Obs^c^ | # Swab | # Infected |
| COWJUN | | 0 | 0 | | 0 | | | 0 | | 0 | | | 0 | | 0 | | | 0 | 0 | 0 | 0 | 0 | 1 | 30 | 1 |
| DOG | | 1 | 28 | | 1 | | | 0 | | 0 | | | 0 | | 0 | | | 0 | 0 | 0 | 0 | 0 | 1 | 0 | 0 |
| GREATD | | 1 | 25 | | 1 | | | 1 | | 25 | | | 0 | | 0 | | | 0 | 0 | 0 | 0 | 0 | 1 | 25 | 2 |
| HH | | 0 | 0 | | 0 | | | 1 | | 30 | | | 0 | | 0 | | | 0 | 0 | 1 | 0 | 0 | 0 | 0 | 0 |
| ING QUAR | | 1 | 24 | | 6 | | | 0 | | 0 | | | 0 | | 0 | | | 0 | 0 | 0 | 0 | 0 | 0 | 0 | 0 |
| MAYHON | | 1 | 0 | | 0 | | | 0 | | 0 | | | 0 | | 0 | | | 0 | 0 | 0 | 0 | 0 | 1 | 25 | 0 |
| P OF P | | 0 | 0 | | 0 | | | 1 | | 24 | | | 2 | | 0 | | | 0 | 0 | 0 | 0 | 0 | 0 | 0 | 0 |
| ROCK 4 | | 1 | 0 | | 0 | | | 0 | | 0 | | | 0 | | 0 | | | 0 | 0 | 0 | 0 | 0 | 1 | 27 | 0 |
| TELLEEN | | 0 | 0 | | 0 | | | 0 | | 0 | | | 0 | | 0 | | | 0 | 0 | 0 | 0 | 0 | 1 | 27 | 0 |
| TRAIN W | | 1 | 0 | | 0 | | | 0 | | 0 | | | 0 | | 0 | | | 0 | 0 | 0 | 0 | 0 | 1 | 26 | 1 |
| U SHAN | | 1 | 32 | | 21 | | | 0 | | 0 | | | 0 | | 0 | | | 0 | 0 | 0 | 0 | 0 | 1 | 0 | 0 |
| 63 FROG | | 1 | 13 | | 0 | | | 0 | | 0 | | | 0 | | 1 | | | 11 | 10 | 0 | 0 | 0 | 0 | 0 | 0 |
| BBT | | 0 | 0 | | 0 | | | 1 | | 25 | | | 6 | | 1 | | | 25 | 8 | 0 | 0 | 0 | 0 | 0 | 0 |
| CHERRY E | | 1 | 0 | | 0 | | | 0 | | 0 | | | 0 | | 1 | | | 0 | 0 | 1 | 23 | 0 | 1 | 27 | 0 |
| CC 3/11 | | 0 | 0 | | 0 | | | 0 | | 0 | | | 0 | | 1 | | | 25 | 24 | 0 | 0 | 0 | 0 | 0 | 0 |
| DONIPH | | 0 | 0 | | 0 | | | 0 | | 0 | | | 0 | | 1 | | | 23 | 4 | 0 | 0 | 0 | 0 | 0 | 0 |
| EGG 4 | | 0 | 0 | | 0 | | | 1 | | 25 | | | 0 | | 1 | | | 22 | 2 | 0 | 0 | 0 | 0 | 0 | 0 |
| **Site Specific Bd Infection Data (number of wetlands = 36), *Cont’d*** | | | | | | | | | | | | | | | | | | | | | | | | | |
|  | | *A. tigrinum* | | | | | | | *A. woodhousii* | | | | | | | *L. catesbeianus* | | | | *L. pipiens* | | | *P. triseriata* | | |
| Site | | Obs. | | # Swab | | # Infected | | | Obs. | | # Swab | | | # Infected | | Obs. | | # Swab | # Infected | Obs. | # Swab | # Infected | Obs. | # Swab | # Infected |
| FANCHER | | 0 | | 0 | | 0 | | | 1 | | 2 | | | 0 | | 1 | | 30 | 1 | 1 | 0 | 0 | 1 | 19 | 0 |
| FLAT11 | | 0 | | 0 | | 0 | | | 0 | | 0 | | | 0 | | 1 | | 29 | 25 | 0 | 0 | 0 | 0 | 0 | 0 |
| FLAT7 | | 0 | | 0 | | 0 | | | 0 | | 0 | | | 0 | | 1 | | 7 | 2 | 0 | 0 | 0 | 1 | 25 | 3 |
| GREEN13 | | 0 | | 0 | | 0 | | | 0 | | 0 | | | 0 | | 1 | | 25 | 23 | 0 | 0 | 0 | 0 | 0 | 0 |
| GREEN3 | | 0 | | 0 | | 0 | | | 0 | | 0 | | | 0 | | 1 | | 24 | 1 | 0 | 0 | 0 | 0 | 0 | 0 |
| IMEL 3 | | 0 | | 0 | | 0 | | | 1 | | 18 | | | 1 | | 1 | | 22 | 19 | 0 | 0 | 0 | 0 | 1 | 0 |
| LAND C | | 1 | | 33 | | 3 | | | 0 | | 0 | | | 0 | | 1 | | 0 | 0 | 0 | 0 | 0 | 0 | 0 | 0 |
| LEGR 4/7 | | 0 | | 0 | | 0 | | | 0 | | 0 | | | 0 | | 1 | | 24 | 5 | 0 | 0 | 0 | 0 | 0 | 0 |
| LETTIE | | 1 | | 13 | | 2 | | | 1 | | 0 | | | 0 | | 1 | | 4 | 1 | 0 | 0 | 0 | 0 | 0 | 0 |
| MARI13 | | 0 | | 0 | | 0 | | | 0 | | 0 | | | 0 | | 1 | | 24 | 24 | 0 | 0 | 0 | 0 | 0 | 0 |
| NEONA | | 0 | | 0 | | 0 | | | 1 | | 28 | | | 0 | | 1 | | 1 | 1 | 0 | 0 | 0 | 1 | 26 | 0 |
| SAWHILL7 | | 0 | | 0 | | 0 | | | 1 | | 24 | | | 1 | | 1 | | 22 | 2 | 0 | 0 | 0 | 0 | 0 | 0 |
| SHARFIN | | 0 | | 0 | | 0 | | | 1 | | 25 | | | 0 | | 1 | | 0 | 0 | 0 | 0 | 0 | 0 | 0 | 0 |
| SRD 9 | | 0 | | 0 | | 0 | | | 0 | | 0 | | | 0 | | 1 | | 26 | 25 | 0 | 0 | 0 | 0 | 0 | 0 |
| STAIN G | | 0 | | 0 | | 0 | | | 0 | | 0 | | | 0 | | 1 | | 23 | 19 | 0 | 0 | 0 | 0 | 0 | 0 |
| STEP | | 0 | | 0 | | 0 | | | 0 | | 0 | | | 0 | | 1 | | 25 | 12 | 0 | 0 | 0 | 0 | 0 | 0 |
| TAD MAD | | 0 | | 0 | | 0 | | | 0 | | 0 | | | 0 | | 1 | | 22 | 5 | 0 | 0 | 0 | 0 | 0 | 0 |
| YELLOWH | | 0 | | 0 | | 0 | | | 1 | | 21 | | | 1 | | 1 | | 10 | 1 | 0 | 0 | 0 | 1 | 27 | 0 |
| TOTALS | | 11 | | 168 | | 34 | | | 13 | | 270 | | | 11 | | 25 | | 424 | 214 | 3 | 23 | 0 | 13 | 285 | 7 |
